# Draft Genome Sequences of a Ceftazidime-Resistant Acinetobacter baumannii Donor and a Conjugal Escherichia coli Recipient with Acquired Resistance

**DOI:** 10.1128/MRA.00024-19

**Published:** 2019-03-28

**Authors:** Hongjie Chen, Xiaoqiong Gu, Charmaine Ng, Laurence Haller, Francis Rathinam Charles, Karina Yew-Hoong Gin

**Affiliations:** a Department of Civil and Environmental Engineering, National University of Singapore, Singapore; b Department of Surgery, National University of Singapore, Singapore; c NUS Environmental Research Institute (NERI), Singapore; University of Arizona

## Abstract

A ceftazidime-resistant Acinetobacter baumannii strain was isolated from hospital wastewater and used as the donor in a filter mating experiment with an Escherichia coli strain as the recipient. Recipient, donor, and transconjugant were sequenced, and both donor and transconjugant were found to harbor highly similar plasmid sequences, suggesting that plasmid transfer had occurred.

## ANNOUNCEMENT

Antibiotic-resistant bacteria (ARB) and antibiotic-resistant genes (ARGs) can act as agents for the selection and transfer of antimicrobial resistance (1–6). To demonstrate the transferability of plasmids containing ARGs from ARB to susceptible strains, a donor ceftazidime-resistant *Acinetobacter baumannii* strain, WPB102, which was isolated from hospital wastewater effluent (3), was selected for the study. A single bacterial colony was heat lysed and amplified using the 16S rRNA gene primers 27F (5′-AGAGTTTGATYMTGGCTCAG-3′) and 1492R (5′-GGYTACCTTGTTACGACTT-3′), followed by identification against the National Center for Biotechnology Information (NCBI) 16S rRNA gene database criterion of at least 99% identity (3). Phenotypic resistance to ceftazidime was confirmed using the AST-GN79 card on a Vitek 2 compact system (bioMérieux). A ceftazidime-susceptible Escherichia coli SCC1 strain (WPB103) was used as the recipient (7). Conjugation was conducted in triplicate via the filter mating method (8). Overnight donor and recipient cultures (optical density at 600 nm [OD_600_] = 0.1) were washed twice and mixed in equal volumes. An aliquot of 100 µl of the mixture was loaded onto a 0.45-µm cellulose nitrate membrane (Sartorius Stedim) and placed onto LB agar for incubation at 37°C for 16 h. Biomass on the membrane was dislodged, serially diluted, and plated on agar supplemented with antibiotics to select for recipient, donor, and transconjugant, namely, chloramphenicol (15 ppm) in tryptone bile X-glucuronide (TBX) agar, ceftazidime (8 ppm) in LB agar, and chloramphenicol (15 ppm) plus ceftazidime (8 ppm) in TBX agar, respectively. The plates were incubated at 37°C for LB agar and 44°C for TBX agar. The transfer efficiency, calculated as the ratio of transconjugants to recipients, was 1.81 × 10^−3^ ± 3.25 × 10^−4^. Antibiogram results from the AST-GN79 panel confirmed that the transconjugant (WPB121) isolated from the selective medium was resistant to ceftazidime.

Total genomic DNA was extracted from single isolates of recipient, donor, and transconjugant using the UltraClean microbial DNA isolation kit (MoBio Laboratories). Genomic DNA was prepared using an NEBNext Ultra DNA library prep kit (New England BioLabs, Inc.) and sequenced on the Illumina HiSeq 4000 platform to obtain 150-bp paired-end reads. Totals of 7.1, 6.8, and 7.1 million paired-end reads were assembled using VelvetOptimiser 2.2.4 (9) with a minimum contig cutoff of 500 bp. The genome coverages for WPB102, WPB103, and WPB121 were 456, 617, and 448× depth, respectively. Contigs were scaffolded with Opera 1.4.1 and finished using FinIS 0.3 (10). The genome sizes for recipient, donor, and transconjugant were 4.6, 3.2, and 4.6 million bp, with G+C contents of 50.7%, 38.8%, and 50.6%, respectively. The genomes contained 67, 85, and 77 contigs, with *N*_50_ values of 188,313, 150,429, and 178,426 bp. Plasmid and chromosome sequences were predicted using PlasFlow 1.1 with default settings (11). Default parameters were used for all software unless otherwise specified.

Plasmid sequences in donor and transconjugant genomes were compared using NCBI BLASTn and were found to share 99% nucleotide similarity and 99% alignment coverage, providing evidence of plasmid-transferred ceftazidime-resistant genes and interspecies transmission of clinically relevant ARGs.

### Data availability.

Whole-genome sequences of the transconjugant, recipient, and donor were deposited in GenBank under the accession numbers CP034426 to CP034428. Raw reads can be found in the NCBI Sequence Read Archive (SRA) under the accession numbers SRR8289913 to SRR8289915.
